# Achieving Impact: Charting the Course to Meet the Challenges Ahead at the 12th International Conference on Typhoid and Other Invasive Salmonelloses

**DOI:** 10.1093/ofid/ofad181

**Published:** 2023-06-02

**Authors:** Jade A Greear, A Duncan Steele, Denise O Garrett

**Affiliations:** Coalition against Typhoid, Sabin Vaccine Institute, Washington, District of Columbia, USA; Enterics, Diagnostics, Genomics and Epidemiology, Global Health, Bill & Melinda Gates Foundation, Seattle, Washington, USA; Coalition against Typhoid, Sabin Vaccine Institute, Washington, District of Columbia, USA

**Keywords:** enteric fever, invasive nontyphoidal salmonella disease, salmonella paratyphi, salmonella typhi, typhoid

## Abstract

Typhoid fever and other invasive salmonelloses remain a major public health concern, primarily in low- and middle-income countries in Asia and Africa, where transmission occurs through contaminated food or water. However, recent developments in research, policy, and implementation offer newfound optimism for prevention and control. Now, more than ever, a coordinated and multisectoral global response is needed. To chart the course to meet the challenges ahead, the Coalition against Typhoid, housed at the Sabin Vaccine Institute, virtually organized the 12th International Conference on Typhoid and Other Invasive Salmonelloses from December 7 to 9, 2021. This commentary provides an overview of the conference's significant findings, highlighting barriers and opportunities for prevention and control. Topics covered include diagnostics advancements, improved data methodologies for a better understanding of the disease burden, the incorporation of environmental surveillance and genomics, the threat of drug resistance, and the use of typhoid conjugate vaccines alongside other integrated solutions.

## Teaser

To strengthen the collective response against typhoid, the Coalition against Typhoid organized the 12th International Conference on Typhoid and Other Invasive Salmonelloses in 2021. This conference brought together researchers, advocates and policymakers aiming to enhance the fight against typhoid. This article describes the topics and research featured at the conference.

‘Enteric fever’ is an umbrella term encompassing typhoid fever and paratyphoid fever, acquired through infection with *Salmonella enterica* serovars Typhi (typhoid fever) and Paratyphi A, B, and C (paratyphoid fever) [1]. For centuries, these infections have caused illness and premature death through contaminated food and water. While typhoid was largely eradicated in the United States and Europe in the 1940s with the arrival of antibiotics and strides in sanitation infrastructure, like the chlorination of water, it remains a daily threat for millions of people living in low- and middle-income countries (LMICs). Globally, there are approximately 14.3 million cases of enteric fever each year, leading to around 133 000 deaths across all ages [1]. Of these cases, at least 11 million are caused by *Salmonella enterica* serotype Typhi alone. Most of those at highest risk are children <15 years of age living in sub-Saharan Africa and South Asia [1]. Invasive nontyphoidal *Salmonella* (iNTS) serovars, such as Typhimurium and Enteritidis, also pose a significant threat, particularly in sub-Saharan Africa, where an estimated 535 000 cases occur annually [2]. The severity of iNTS in sub-Saharan Africa can be attributed to a high concentration of vulnerable groups such as the elderly, malnourished infants, and individuals with compromised immune systems from diseases like sickle-cell, human immunodeficiency virus (HIV), and malaria [2].

Together, these illnesses result in a substantial burden of disease, including an annual loss of 8.4 million (95% uncertainty interval, 4.7–13.6 million) disability-adjusted life years (DALYs) [1]. LMICs bear a greater impact on morbidity, mortality, and financial costs [3]. Socioeconomic determinants like access to water and sanitation infrastructure, severity of exposure to climate crises like flooding and drought, health literacy rates and consumption patterns, all exacerbate the spread of these diseases through contaminated food and water sources [3].

On December 22, 2017, the World Health Organization (WHO) prequalified and recommended the first typhoid conjugate vaccine (TCV), which offers long-lasting immunity, requires only one dose and can be given to children as young as six months of age. This makes it suitable for use in areas where typhoid is prevalent [4]. Subsequently, in April 2018, Gavi opened a US $85 million funding window to support TCV introduction in eligible countries, creating a route to increase access to the vaccine where it is needed most [5]. With this new support, in November 2019, Pakistan became the first country in the world to introduce TCV into its routine immunization program—in part to help stem a 3-years-long outbreak of extremely drug-resistant typhoid in Sindh Province [6]. Through 3 phases of mass immunization campaigns across 3 years, Pakistan has vaccinated more than 30 million children with TCV. Since Pakistan's phase 1 introduction campaign, other national introductions have been launched in Liberia, Zimbabwe, Samoa, Nepal, and Malawi.

However, vaccines alone are not sufficient. TCVs are a promising addition to an integrated package of solutions to combat endemic typhoid, but other critical interventions must also be addressed. Investments in water, sanitation, and hygiene (WASH) infrastructure are more important than ever, particularly as climate change alters the frequency of disease. Curbing the global spread of antimicrobial resistance (AMR) in *Salmonella* bacterial strains must become a priority as the threat of pre-antibiotic era mortality rates looms near [6]. In 2017, the World Health Organization published a list of priority bacteria for which the development of antibiotics is critically needed, categorizing *Salmonellae* as high priority for its fluoroquinolone resistance [7]. With limitations in diagnosing typhoid in low-resource settings, true burden is difficult to capture, and presumed illness is often treated with unnecessary antibiotics, which amplifies the risk of AMR. Neither paratyphoid nor iNTS have available vaccines and so must rely solely on infrastructural measures of mitigation. Further, introducing a vaccine into a country’s routine immunization program requires buy-in and collaboration from government and societal partners weighing several competing priorities – a notable challenge over the last three years as the world came together to address the COVID-19 pandemic.

Now, more than ever, coordinated and multisectoral global response is needed to take on typhoid effectively. To chart the course to meet the challenges ahead, the Coalition against Typhoid, housed at the Sabin Vaccine Institute, supported by the Bill & Melinda Gates Foundation, convened the 12th International Conference on Typhoid and Other Invasive Salmonelloses from 7–9 December 2021. For the first time in conference history more than 450 researchers, policy makers, immunization managers and advocates from across 46 countries were convened through a full-scale virtual event. Over 70% of the delegates were participating from Asia and Africa, including many from typhoid-endemic countries. The event created space to share and discuss research findings, exchange ideas, and identify the best strategies to reduce the burden of typhoid, paratyphoid and iNTS for communities around the world.

In this supplement to *Open Forum Infectious Diseases*, we present a comprehensive overview of the major themes discussed at the conference. These themes include diagnostics, data and surveillance, drug resistance, vaccines, and other prevention and control interventions. The articles in this supplement delve into these topics in more detail, providing a deeper understanding of the advancements and challenges discussed during the conference.

## DIAGNOSTICS FOR TYPHOID

Accurate diagnosis of typhoid, paratyphoid and iNTS is crucial for timely treatment and appropriate medical care. However, there are many barriers to achieving accurate diagnosis in areas where these illnesses are endemic.

The WHO currently recommends a blood culture isolation of *S*. Typhi as the gold standard for diagnosing typhoid fever [8]. However, blood culture is not generally accessible or feasible in low-resource, high-burden areas due to its high cost, including the expenses associated with equipment, reagents and the specialized personnel and laboratories necessary to perform them. Further, even when blood culture tests are performed, they only identify approximately 59% (95% confidence interval: 54%–64%) of typhoid cases correctly [9]. The less expensive and commonly used diagnostic tool in endemic settings is the Widal test, an agglutination, serological test that has been used for over 100 years ago but is considered unreliable for diagnosis of typhoid fever since false positive and negative results are common [10].

In many instances, the lack of access and reliability of available diagnostic tools leaves diagnosis on a patient's differential clinical presentation, which is complicated by the many fever-presenting illnesses in these regions such as malaria, pneumonia, influenza, and other febrile illnesses. Without accurate and rapid diagnostic tests, many countries lack the ability to recognize and intervene to control typhoid outbreaks. Furthermore, asymptomatic carriers of *S.* Typhi remain challenging to detect and treat, leading to the possibility of continuous low-grade transmission in the community [11]. These challenges illustrate the vital need for more accurate and rapid diagnostic tests, a need echoed throughout several presentations at the conference and throughout the global health community. Gavi, recognizing this issue for effective typhoid control, has initiated landscaping to understand the market and optimal assays for early detection [12]. In this supplement, Sapkota, FIND, the global alliance for diagnostics, et al. expand on this by exploring existing and future techniques for diagnosing typhoid that meet the standards for sensitivity, specificity, speed, and cost-effectiveness in the local setting.

## DATA METHODOLOGY AND ACCESS TO UNDERSTAND BURDEN

A comprehensive set of data on typhoid burden is vital to helping policymakers prioritize investment in prevention and control strategies. However, many countries lack the diagnostic and surveillance infrastructure to provide this evidence, resulting in data gaps. The impact of having quality data to inform decision making can be seen across the last several years of typhoid control efforts, particularly through four landmark studies: Surveillance of Enteric Fever in India (SEFI) [13], the Surveillance for Enteric Fever in Asia Project (SEAP) [14], the Severe Typhoid Fever Surveillance in Africa (SETA) program, and the Strategic Typhoid Alliance across Asia and Africa (STRATAA) [15]. With these studies expanding the library of local, national, and regional surveillance data in typhoid-endemic areas, we gained a greater understanding of the global incidence of enteric fever [1]—an understanding that has directly influenced the early introduction of TCV, reaching millions of children with lifesaving vaccines, which is even more remarkable in that it mostly occurred during the global pandemic. However, these large studies are long, expensive, and resource intensive, leaving remaining data gaps for countries unable to follow suit and hindering further advancements in control and prevention strategies.

Innovation in methodology to understand typhoid burden in resource-limited settings is imperative and was the subject of several presentations at the 12th International Conference. One such solution, serological surveillance or serosurveys, was described in a symposium lead by Jessica Seidman, Sabin Vaccine Institute, and Jason Andrews, Stanford University. These tests estimate levels of background infection, providing a more comprehensive picture of the true spread of typhoid disease within a community and enabling the comprehensive assessment of typhoid burden to inform vaccine introduction. In this supplement, Kristen Aiemjoy (UC Davis) expands on the role of serological tools in nonroutine clinical use cases, including population-based estimation of typhoid incidence, outbreak identification, and confirmation of recent typhoid infection in intestinal perforations cases. There is also a potential role for serological tools in evaluating vaccine impact on community transmission of typhoid by seeing whether, over time after vaccine introduction, the population level of anti-HlyE antibodies declines, indicating less typhoid transmission in a community.

Typhoid intestinal perforations (TIPs) are a severe, life-threatening complication of typhoid. In addition, they can be used as a surrogate indicator for typhoid that would allow governments to make decisions on TCV introduction in areas without blood culture capability. Megan Birkhold, University of Maryland School of Medicine, discusses this and overviews the epidemiology, clinical presentation, and time trends of TIP in 6 SETA countries.

With increasing antimicrobial resistance, whole-genome sequencing, commonly used for infectious disease epidemiology in several high-income countries, can also be of great use for understanding the emergence and spread of drug-resistant typhoid. Although in low-resources settings, bioinformatics capacity is often a limiting factor in the implementation of genomic epidemiology. Novel tools and programs such as Typhi Pathogenwatch, TyphiNet, SEQAFRICA, and the Africa Centers for Disease Control's Pathogen Genomics Initiative are working diligently to address these barriers by creating accessible platforms and services to support the generation, analysis, and visualization of *Salmonella* Typhi genomic data. In this supplement, and as presented during the conference, Megan Carey, University of Cambridge, details these platforms and tools, highlights opportunities to access training, data, and visualization tools, and outlines key remaining challenges.

## DRUG RESISTANCE: AN UNPRECEDENTED THREAT TO GLOBAL HEALTH

Antibiotics have been considered the only effective treatment for enteric fever and iNTS since the 1940s. However, new evidence shows that even newer antibiotics that were once effective against typhoid are now becoming less effective, particularly in low-resource areas where diagnosis and appropriate treatment is challenging. The emergence of 2 predominant forms of resistance—extensively drug-resistant (XDR) typhoid in Pakistan and multidrug-resistant typhoid, which is widespread in Africa—suggests that currently used and WHO-recommended antimicrobials may not be able to successfully treat typhoid for much longer [16]. Fortunately, TCVs have the potential to be an effective tool against antimicrobial resistance (AMR) by preventing new cases from arising, and over the next ten years, have the potential to avert two-thirds of drug-resistant typhoid cases, disability-adjusted life years (DALYs), and deaths [16]. Further, within and outside of the scope of enteric fever, addressing rapidly rising rates of AMR has become a global health priority [17].

While TCVs are promising, interim treatment strategies are necessary for new typhoid cases until widespread TCV coverage is achieved. As presented at the conference, and detailed in this supplement, Christopher Parry, Liverpool School of Tropical Medicine, and et al. outline the strength of the existing evidence supporting recommendations for the treatment of typhoid fever. This includes acute disease, extensively drug-resistant typhoid, as well as the rationale for and use of antimicrobial combinations. Additionally, the manuscript provides an update about available old and new antimicrobials that may be used to address the emerging resistance problem.

## TYPHOID CONJUGATE VACCINES AND AN INTEGRATED PACKAGE OF SOLUTIONS FOR ENTERIC FEVER CONTROL

The power of translating data and engaging decision makers with evidence has been heavily reflected in the policy-driven milestones of the past decade. This is seen in the 2017 WHO SAGE policy recommendation to introduce TCV in the routine immunization programs of typhoid-endemic countries, leading to the WHO's formal adoption in 2018. With this endorsement from WHO, in April 2018, Gavi, the Vaccine Alliance opened a US $85 million funding window to support TCV introduction in eligible countries, creating a route to increase access to the vaccine where it is needed the most. Understanding cost and policy in decision making for vaccine introduction was a focal point of the 12th International Conference. In this supplement, Allyson Russell, Gavi, the Vaccine Alliance, and et al. describe Gavi’s investment and support for countries that are in the process of introducing TCV and Gavi’s view on how the TCV program is expected to grow in the post-acute phase of the COVID-19 pandemic.

The introduction of, and increasing access to, these lifesaving vaccines marks a paramount achievement in typhoid control and prevention. Already, exciting data showcasing the benefits of these vaccines has been published. A recent field evaluation of Typbar-TCV in Sindh, Pakistan, demonstrated 98% effectiveness of the vaccine against culture-confirmed non-XDR *Salmonella* Typhi [18], while analyses from Hyderabad revealed 97% effectiveness against XDR cases [19]. However, we still have far to go in ensuring these vaccines reach all those at risk. The study by Nginache Nampota-Nkomba, Kamuzu University of Health Sciences, et al. discusses the body of data on the effectiveness of TCVs based on evidence collected in Africa and Asia and additional potential for TCVs to reduce disease burden in these regions. As presented at the conference, they also make recommendations on what still needs to be done to reduce the health equity gap with typhoid fever.

We know that for the greatest impact, vaccines must be introduced in tandem with other prevention measures like water, sanitation and hygiene (WASH) improvements. The collective strategizing of the future of typhoid and iNTS control was central to this year's conference, and a prompt that inspired several posters and presentations. In this supplement, Jessie Chen, Bill & Melinda Gates Foundation, et al. details an integrated approach to achieve impact. They also highlight data blind spots and knowledge gaps that need to be addressed as part of our efforts to control and eliminate typhoid.

## BEYOND TYPHOID

With all the exciting progress in typhoid prevention and control, we must not turn a blind eye to paratyphoid and iNTS. Infection with other *Salmonella enterica* serovars, Paratyphi A, B, and C, lead to approximately 3.4 million cases of paratyphoid fever each year across all ages [1]. In this supplement, Calman MacLennan, Bill & Melinda Gates Foundation, explores the case for *Salmonella* combination vaccines, presenting the current state of the pipeline of these vaccines as monovalent or bivalent products. However, as indicated above, we need evidence that allows us to characterize and understand the burden and impact of an illness before designing effective strategies to take it on. The manuscript by Senjuti Saha, Child Health Research Foundation, et al. provide an overview of a genomic surveillance method for *Salmonella* Paratyphi A, contributing to our greater understanding of the pathogen and the identification of better development of interventions to fight it. With an estimated 535 000 cases of iNTS globally each year across all ages, and rising, research for a broad-spectrum iNTS vaccine and investigation of other solutions are also under way [1]. One project highlighting the development of a trivalent *Salmonella* vaccine that could protect against typhoid and 2 types of iNTS was presented at the conference by Myron Levine, University of Maryland School of Medicine.

The 12th International Conference introduced three roundtable discussion groups, where a curated group of experts engaged in moderated and interactive conversations to address key challenges, strategies and recommend solutions. One of the roundtables focused on iNTS, and a manuscript by John Crump, University of Otago, et al. summarizes the session’s themes, covering burden, epidemiology, diagnosis, treatment, and vaccines for iNTS.

## CONCLUSIONS

Over the course of three days, the 12th International Conference on Typhoid and Other Invasive Salmonelloses provided a virtual platform for researchers, experts, and advocates to share and discuss new research findings, exchange ideas, and identify the strategies to reduce the burden of typhoid, paratyphoid and iNTS diseases worldwide. In closing, the Bill & Melinda Gates Foundation provided valuable insights into the future of typhoid control, highlighting new evidence, ideas, and tools at our disposal. Despite the challenges posed by the COVID-19 pandemic, the conference demonstrated our collective perseverance and dedication to advancing prevention strategies and research for enteric fever and iNTS. The event served as a catalyst for applying lessons learned and propelling us into the next generation of enteric fever control. This conference signifies our commitment to addressing the difficulties presented by these diseases and our determination to forge ahead. Building upon this success, the 13th International Conference on Typhoid and Other Invasive Salmonelloses will take place from December 5–7, 2023, in Kigali, Rwanda.
